# Molecular characterization of *Fusarium venenatum*-based microbial protein in animal models of obesity using multi-omics analysis

**DOI:** 10.1038/s42003-024-05791-9

**Published:** 2024-01-26

**Authors:** Daniel Junpyo Lee, An Na Kang, Junbeom Lee, Min-Jin Kwak, Daye Mun, Daseul Lee, Sangnam Oh, Younghoon Kim

**Affiliations:** 1 Department of Agricultural Biotechnology and Research Institute of Agriculture and Life Science, Seoul National University, Seoul, 08826 Korea; 2 Agricultural Microbiology Division, Department of Agricultural Biology, National Institute of Agricultural Sciences, Wanju-gun, 55365 Korea; 3 Department of Functional Food and Biotechnology, Jeonju University, Jeonju, 55069 Korea

**Keywords:** Biomaterials - proteins, Applied microbiology

## Abstract

Microbial protein, produced by fermentation of *Fusarium venenatum* is a promising candidate alternative protein source. Previous study has demonstrated its ability to improve hyperlipidemia in rats, yet the related mechanism remains unclear. In this study, we aimed to evaluate the potential of *F. venenatum* as an alternative protein source and its impact on lipid metabolism using multi-omics analysis. Initial experiments with *Caenorhabditis elegans* revealed that *F. venenatum* enhanced longevity, improved immune responses, and reduced lipid metabolism by downregulating fat synthesis-related genes. Subsequently, we conducted experiments with mice on a high-fat diet to confirm the anti-obesity effects of *F. venenatum*. The groups fed *F. venenatum* showed improved lipid profiles and reduced hepatic fat accumulation. Furthermore, fecal metabolomic analysis showed higher excretion of primary bile acid and cholesterol in the groups fed *F. venenatum* which might lead to a decrease in lipid digestion and hepatic fat accumulation. Collectively, this series of experiments revealed the potential of *F. venenatum* as a sustainable alternative protein and its application as an anti-obesity supplement.

## Abstract (web summary)

*Fusarium venenatum* is a potential protein source in *Caenorhabditis elegans* and mice that exhibits anti-obesity properties by improving lipid profiles and reducing fat accumulation.

## Introduction

Obesity has become a global epidemic. Obesity has increased in several countries worldwide, with more than 600 million adults reported as obese and more than 1.9 billion as overweight^1^. Obesity increases the risk of chronic diseases such as type II diabetes, coronary heart disease, and cancer^2^. In recent days, several studies have focused on the anti-obesity effect of gut microbial modulation agents, including probiotics, prebiotics, and live gut microbial organisms.

Microbial protein, produced by fermentation of *Fusarium venenatum*, which is a fungal species that can produce mycoproteins, contains high protein, high fiber, low fat, and relatively low energy^3^. It can produce all essential amino acids and has a protein digestibility-corrected amino acid score (PDCAAS) of 0.996, which is higher than that of chicken and beef, and it is close to that of egg and milk^4^. In addition, a previous study demonstrated that the bioavailability of amino acids in *F. venenatum* was higher than that in plant proteins and was close to that in milk^5^. In 2010, Ruxton and McMillan demonstrated that the rich fiber contents of *F. venenatum* could help to improve the blood lipid profile by increasing high-density lipoprotein (HDL) and decreasing low-density lipoprotein (LDL) and total cholesterol (TC)^6^. An in vivo study using a Triton X-100-induced hyperlipidemic rat model also showed that intake of *F. venenatum* significantly reduced the plasma levels of TC, triglycerides (TG), and LDL compared to controls^7^. In addition, an in vitro experiment using *F. venenatum* showed that the structure of *F. venenatum* may entrap digestive enzymes such as amylase, lipase, and bile salts, which may lead to a decrease in starch hydrolysis and lipolysis^8^. These studies proposed that *F. venenatum* could help to improve the lipid profile; however, there were no studies related to *F. venenatum* as an anti-obesity supplement.

In this study, we aimed to evaluate *F. venenatum*-based microbial protein as an alternative protein source and as a functional food to treat obesity. We conducted two in vivo experiments using *Caenorhabditis elegans* and C57BL/6 mice. In *C. elegans* experiment, we investigated the effect of *F. venenatum* on lifespan, immune response, and fat metabolism. Generally, the nematode *C. elegans* is commonly used as an experimental model with several advantages, including cost-effectiveness, ease of handling, simple genetics, amenability to high-throughput screening, and a transparent body. Although the fat regulation in *C. elegans* is different from that in mammals, the important metabolic pathways related to fat metabolism found in mammals are highly conserved in *C. elegans*, including fatty acid synthesis, elongation desaturation, and β-oxidation^9^. Next, we investigated the anti-obesity effect of *F. venenatum* in mice experiments by modulation of obesity-related indicators and intestinal microbiota composition.

## Results

### Nutritional composition and mycotoxin quantification assay of *Fusarium venenatum*

The nutritional composition of *F. venenatum* KACC No.49797 (A3/5) is shown in Supplementary Table 1, and the mycotoxin quantification assay revealed that *F. venenatum* produces low levels of mycotoxins, as shown in Supplementary Table 2. Fumonisins B1 was found at a level of 8.60 µg/kg, while fumonisins B2, zearalenone, and deoxynivalenol were not detected. According to the U.S. Food and Drug Administration, the maximum allowable levels for total fumonisins in human foods are 2–4 mg/kg, while for animal feeds, they range from 5 to 100 mg/kg^10^. The detected level of 8.60 µg/kg for fumonisins B1 in *F. venenatum* is lower than established limits.

### Evaluation of longevity and immune response of *F. venenatum* using *C. elegans*

**Fig. 1:**
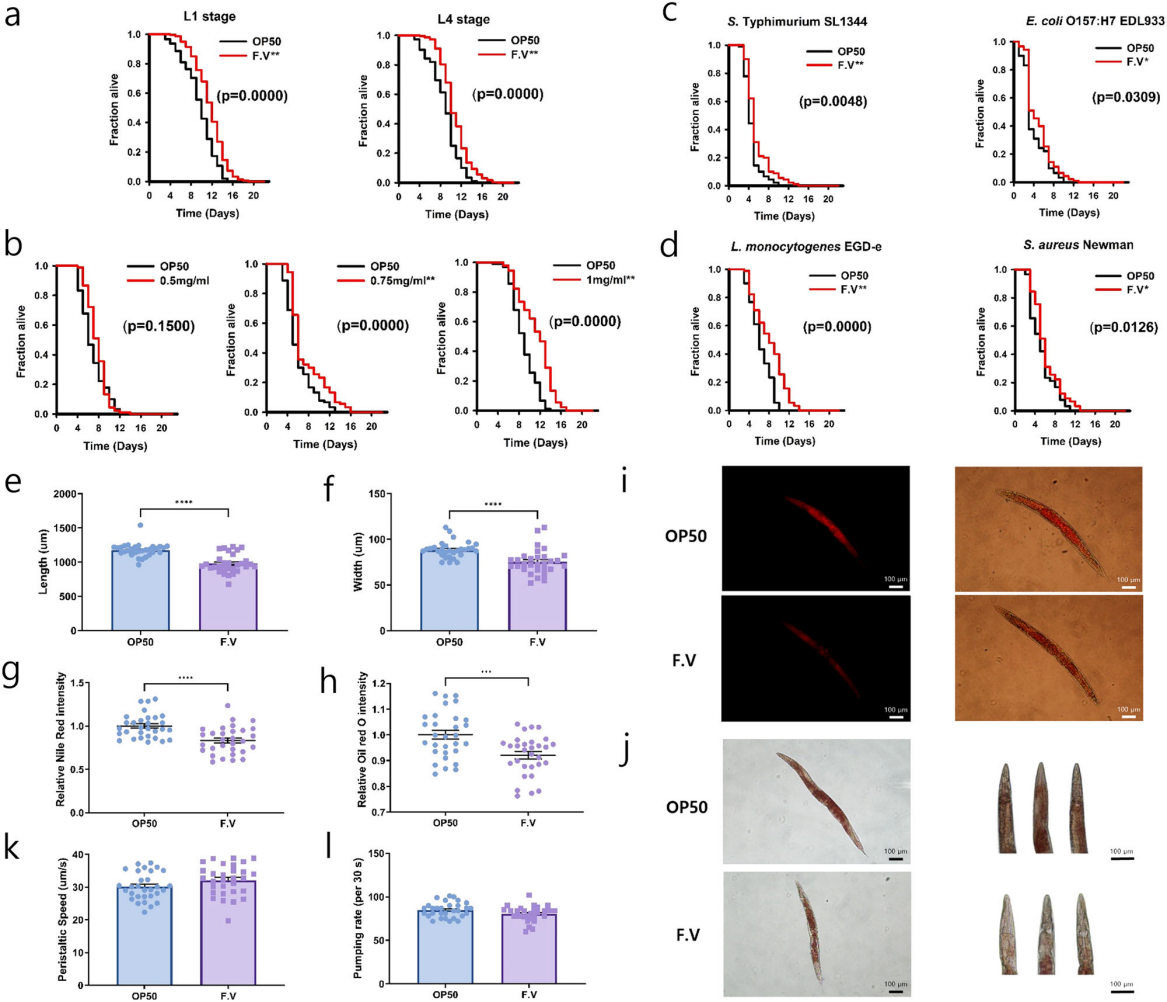
*F. venenatum* prolonged lifespan, enhanced immune response, and reduced fat accumulation in *C. elegans*. **a** Lifespan assay of *C. elegans* feeding *F. venenatum* starting from the L1 and L4 stages. **b** Lifespan assay of *C. elegans* feeding extracted protein from *F. venenatum* (0.5, 0.75, and 1 mg/mL). **c** Killing assay with gram-negative bacteria. **d** Killing assay with gram-positive bacteria. **e** Length of *C. elegans*. **f** Width of *C. elegans*. **g** Quantification of Nile red intensity. **h** Quantification of Oil red O intensity. **i** Nile red stained *C. elegans* (left) and merged with bright field (right). Scale bar, 100 µm. **j** Whole-body image of Oil red O stained *C. elegans* (left) and head image of Oil red O stained *C. elegans* (right). Scale bar, 100 µm. **k** Pumping rate of *C. elegans*. **l** Peristaltic speed of *C. elegans*. Data are expressed as means ± SEM. Statistical analysis was performed compared to OP50 using the Kaplan–Meier method in (**a**–**d**). The differences were considered significant when *p*-value was below 0.05 (*) and 0.01 (**). Statistical analysis was performed using *t*-test in (**e**–**h**), (**k**), and (**l**). The differences were considered significant when the *p*-value was below 0.05 (*), 0.01 (**), 0.001 (***), and 0.0001 (****). OP50 group, received *E. coli* OP50; F.V group, received *E. coli* OP50 with *F. venenatum*.

We first evaluated whether *F. venenatum* affected the lifespan of *C. elegans* L1 (larval) and L4 (young adult) stages. The group of *C. elegans* fed *E. coli* OP50 was referred to as OP50, while the group fed *E. coli* OP50 + *F. venenatum* (20 mg/mL) was referred to as the F.V. The F.V group showed a significantly extended lifespan at both L1 and L4 stages compared with the OP50 group (Fig. 1a and Supplementary Table 3) (*p* = 0.0000 for L1 and *p* = 0.0000 for L4).

Subsequently, we investigated the effect of extracted proteins from *F. venenatum* on the lifespan of *C. elegans*, and we confirmed that extracted protein concentrations of 0.75 and 1 mg/mL significantly enhanced the lifespan of *C. elegans* compared to the OP50 (*p* = 0.0000 for 0.75 mg/mL and *p* = 0.0000 for 1 mg/mL). The protein concentration of 0.5 mg/mL improved the lifespan of *C. elegans* but did not show any significant difference with the OP50 group (Fig. 1b) (*p* = 0.1500). These results indicate that both intact *F. venenatum* and extracted protein concentrations can increase the lifespan of *C. elegans* in vivo.

A killing assay was conducted to assess whether feeding *F. venenatum* enhances the host’s immune response against pathogenic bacteria. The F.V group showed a significant protective effect against *S*. Typhimurium SL1344 and *E. coli* O157:H7 EDL933 compared to the OP50 group (Fig. 1c and Supplementary Table 3) (*p* = 0.0048 for *S*. Typhimurium SL1344 and *p* = 0.0309 for *E. coli* O157:H7 EDL933). Additionally, the viability of *C. elegans* in the F.V group was significantly enhanced compared to that in the OP50 group when *L. monocytogenes* EGD-e, and *S. aureus* Newman were used as pathogenic bacteria (Fig. 1d) (*p* = 0.0000 for *L. monocytogenes* EGD-e and *p* = 0.0126 for *S. aureus* Newman). Collectively, these results indicate that *F. venenatum* improves the host’s immune response against both gram-negative and gram-positive bacteria.

### Characteristics of *C. elegans* after exposure to *F. venenatum*

Body size was measured to evaluate whether feeding *F. venenatum* change the phenotype of *C. elegans*. After an exposure period of 48 h, the F.V group showed significantly decreased length and width compared to the OP50 group (Fig. 1e, f) (*p* < 0.0001 for length and *p* < 0.0001 for width). Reduced body size may be due to reduced fat accumulation. Therefore, Nile red and Oil Red O staining were conducted to reveal the effect of *F. venenatum* on fat regulation. Nile red staining revealed significantly lower fat accumulation in the F.V group compared to the OP50 group (Fig. 1g, i) (*p* < 0.0001). A similar result was observed in Oil Red O staining (Fig. 1h, j) (*p* = 0.0006). However, the peristaltic speed (energy expenditure) and pumping rate (feed intake) of worms were not different between the OP50 and F.V groups (Fig. 1k, l) (*p* = 0.0967 for peristaltic speed and *p* = 0.0573 for pumping rate). Collectively, these results indicated that reduced fat accumulation by *F. venenatum* is unlikely to be caused by changes in *C. elegans* behavior.

### Multi-omics analysis of *C. elegans* after exposure to *F. venenatum*

**Fig. 2:**
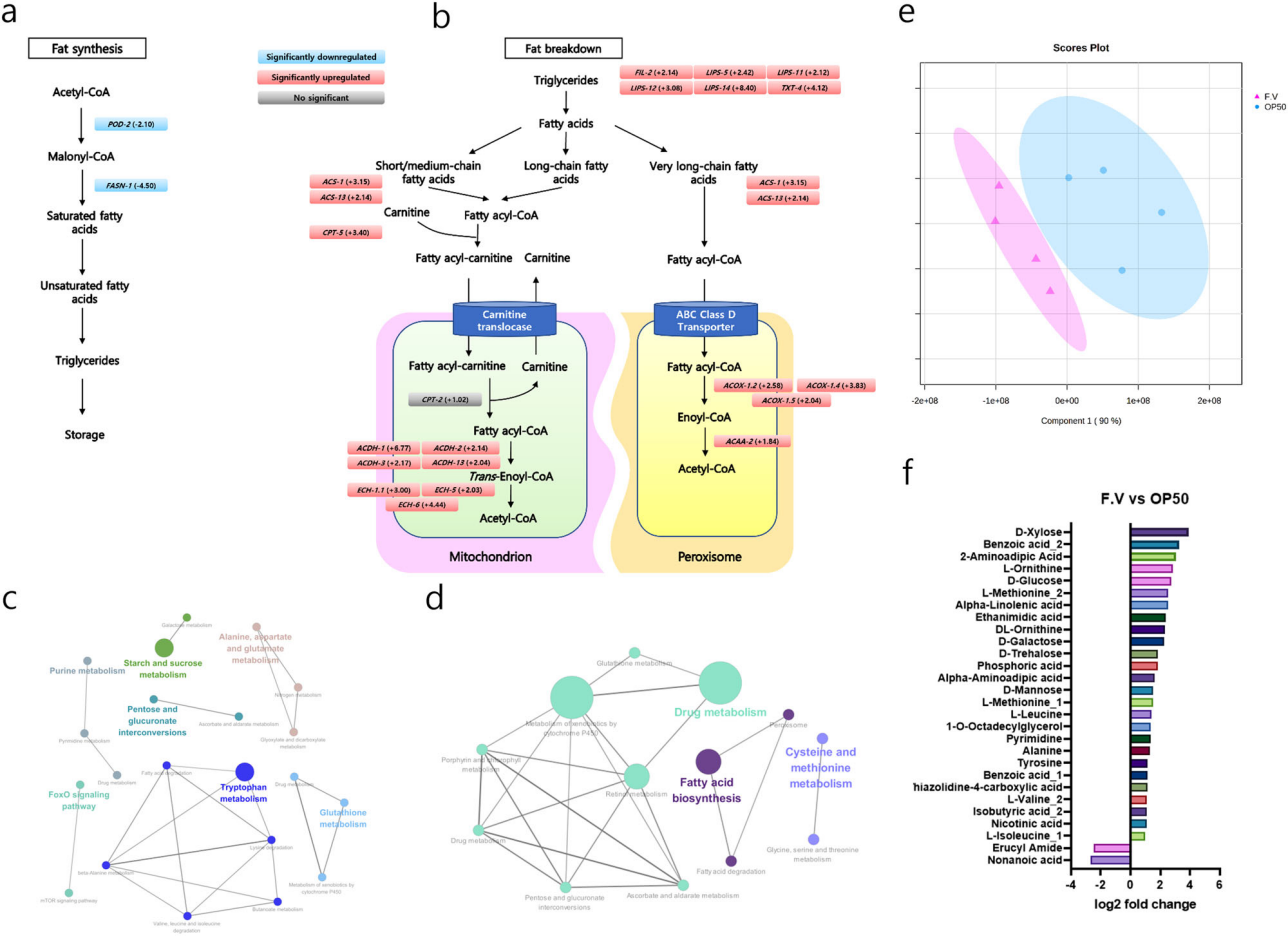
*F. venenatum* altered gene expression related to fat metabolism and metabolites composition in *C. elegans*. **a** Significantly downregulated genes related to the fat synthesis pathway in F.V compared to OP50. **b** Significantly upregulated genes related to fat breakdown pathway in F.V compared to OP50. **c** Upregulated pathway via feeding *F. venenatum*. The genes with a threshold value of fold change > 2 and *p*-value < 0.05 in F.V compared to OP50 were used. **d** Downregulated pathway via feeding *F. venenatum*. The genes with a threshold value of fold change < −3.5 and *p*-value < 0.05 in F.V compared to OP50 were used. **e** PLS-DA analysis with metabolites of F.V and OP50. **f** Upregulated metabolites via feeding *F. venenatum*. The metabolites with a threshold value of fold change > 2 in F.V compared to OP50 were used. The differences were considered significant when *p*-value was below 0.05 (*). OP50 group, received *E. coli* OP50; F.V group, received *E. coli* OP50 with *F. venenatum*.

A whole-transcriptomic analysis was performed to identify changes in fat metabolism by feeding *F. venenatum*. The genes *POD-2* (fold change −2.10) and *FASN-1* (fold change −4.50), which are related to the fat synthesis pathway, were significantly downregulated upon feeding *F. venenatum* (Fig. 2a) (*p* = 0.0000 for *POD-2* and *p* = 0.0000 for *FASN-1*). Conversely, a large number of genes associated with mitochondria and peroxisome fatty acid breakdown pathways were significantly upregulated upon feeding *F. venenatum* (Fig. 2b). The genes that were significantly upregulated upon feeding *F. venenatum* were used to map the upregulated pathways (Fig. 2c). We found that the Foxo signaling pathway, tryptophan metabolism, and glutathione metabolism, were upregulated, and fatty acid biosynthesis, cysteine and methionine metabolism, and drug metabolism were downregulated (Fig. 2d). Collectively, these findings suggested that *F. venenatum* may improve longevity and inhibit fat storage by downregulating the fat synthesis pathway and upregulating the fat breakdown pathway.

Metabolomic analysis was performed to identify altered metabolite profiles by feeding *F. venenatum*. PLS-DA analysis showed differences in metabolite profiles between the OP50 and F.V groups (Fig. 2e). Metabolites that exhibited more than twofold changes between the two groups are shown in Fig. 2f. Twenty-seven metabolites, including d-xylose, 2-aminoadipic acid, alpha-linolenic acid, nicotinic acid, and branched-chain amino acids (BCAAs), were upregulated, while two metabolites were downregulated by more than twofold in the F.V group compared to that in the OP50 group.

### Growth performance of mice fed *F. venenatum*

**Fig. 3:**
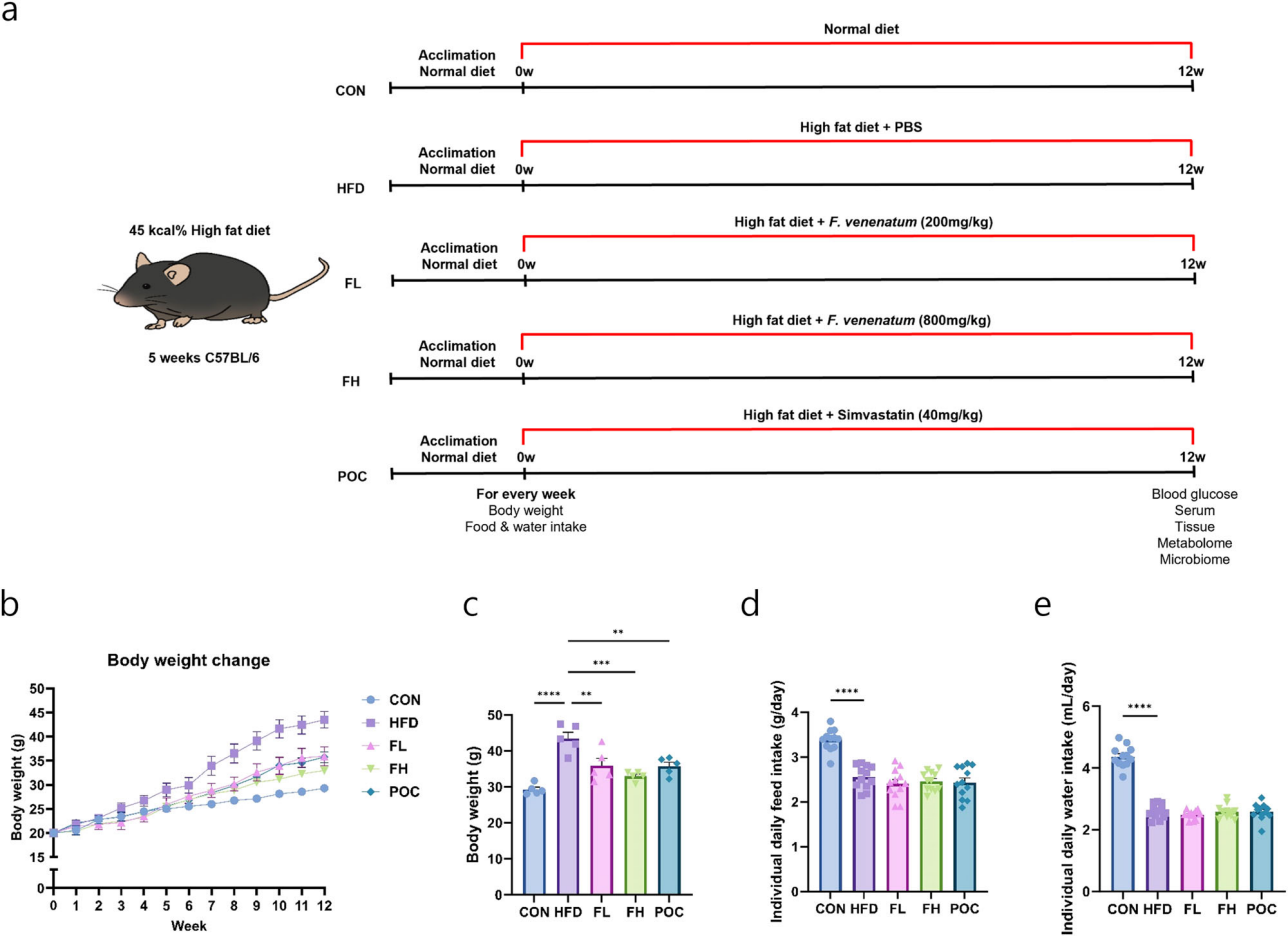
*F. venenatum* reduced body weight gain of high-fat diet-fed mice with no significant difference in feed and water intake. **a** Experimental design. **b** Body weight change. **c** Body weight on week 12. **d** Individual daily feed intake. **e** Individual daily water intake. Data are expressed as means ± SEM. Statistical analysis was performed using One-way ANOVA, and differences were considered significant when the *p*-value was below 0.05 (*), 0.01 (**), 0.001 (***), 0.0001 (****). CON group received no oral administration with a normal diet; HFD group, received an oral administration of 200 µL of PBS with a high-fat diet; the FL group received an oral administration of 200 mg/kg of *F. venenatum* with a high-fat diet; the FH group, received an oral administration of 800 mg/kg of *F. venenatum* with a high-fat diet; POC group, received an oral administration of 40 mg/kg of simvastatin with a high-fat diet.

The overall experimental design is illustrated in Fig. 3a. After 12 weeks, the HFD group showed significantly heavier body weights compared to the other groups (Fig. 3b, c) (*p* < 0.0001 for CON, *p* = 0.0055 for FL, *p* = 0.0002 for FH, and *p* = 0.0044 for POC). Daily feed intake was significantly increased in the CON group compared to the other groups, possibly due to the calorie difference between normal chow and a high-fat diet (*p* < 0.0001). However, there was no significant difference in the daily feed intake between the groups fed with a high-fat diet (Fig. 3d). Daily water intake showed a similar trend (Fig. 3e). Collectively, the results indicated that *F. venenatum* reduced body weight with no difference in feed and water intake.

### Serum analysis of mice fed *F. venenatum*

**Fig. 4:**
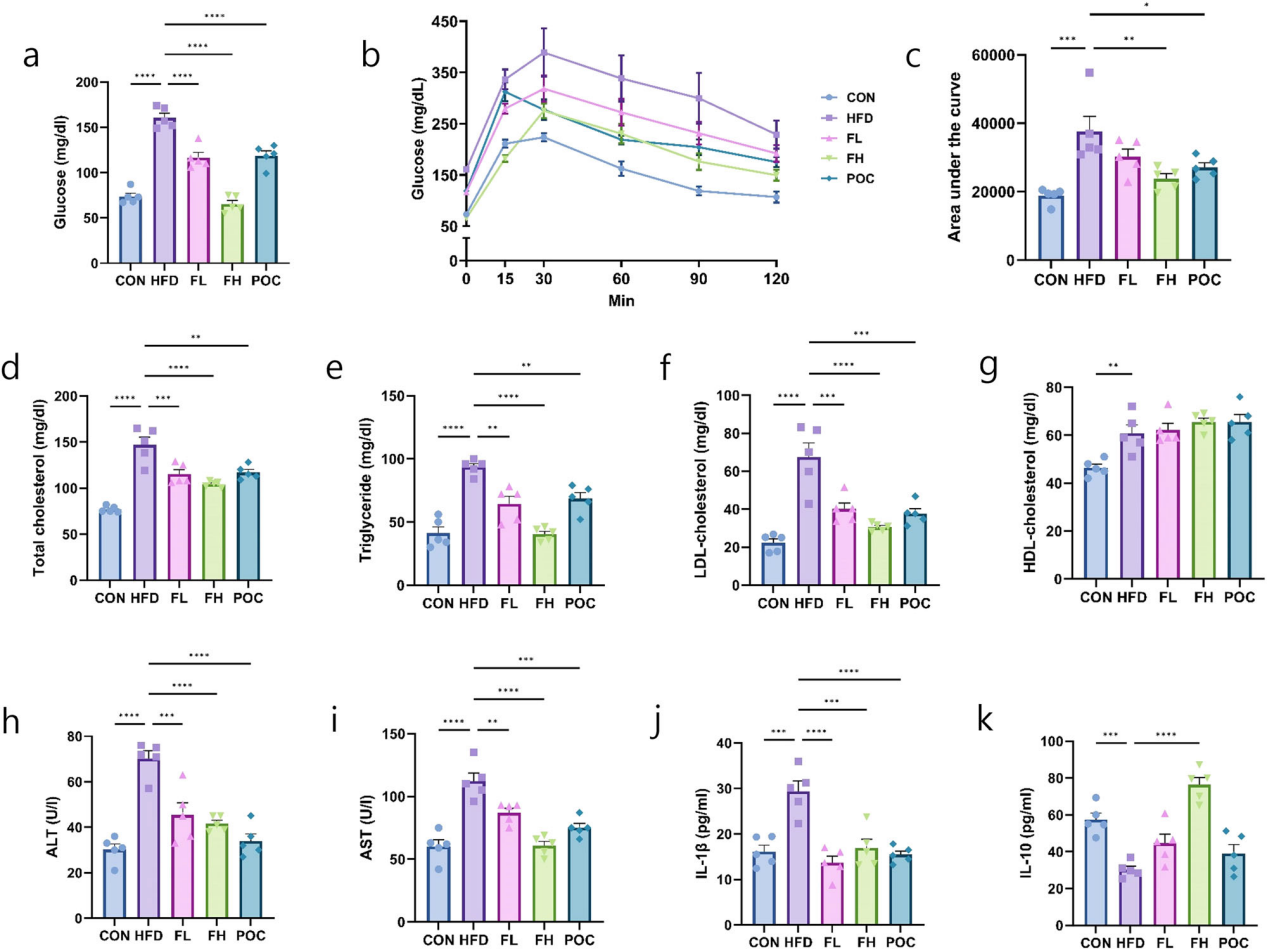
*F. venenatum* attenuated hyperglycemia and improved lipid profile in the serum of high-fat diet-fed mice. **a** Fasting blood glucose on week 12. **b** Blood glucose level on oral glucose tolerance test (OGTT). **c** Area under the curve. **d** Total cholesterol. **e** Triglyceride. **f** Low-density lipoprotein–cholesterol. **g** High-density lipoprotein–cholesterol. **h** Alanine transaminase. **i** Aspartate transaminase. **j** IL-1β. **k** IL-10. Data are expressed as means ± SEM. Statistical analysis was performed using One-way ANOVA, and differences were considered significant when the *p*-value was below 0.05 (*), 0.01 (**), 0.001 (***), 0.0001 (****). CON group received no oral administration with a normal diet; the HFD group received an oral administration of 200 µL of PBS with a high-fat diet; FL group received an oral administration of 200 mg/kg of *F. venenatum* with a high-fat diet; FH group received an oral administration of 800 mg/kg of *F. venenatum* with a high-fat diet; POC group received an oral administration of 40 mg/kg of simvastatin with a high-fat diet.

At week 12, mice were fasted for 16 h before the fasting blood glucose levels were confirmed. Compared with the HFD group, the CON, FL, FH, and POC groups showed significantly reduced blood glucose levels by approximately 54, 27, 59, and 26%, respectively (*p* < 0.0001, *p* < 0.0001, *p* < 0.0001, and *p* < 0.0001, respectively). The serum glucose levels of mice in the FH and CON groups had no significant difference (Fig. 4a) (*p* = 0.7421). After confirming fasting blood glucose levels, an oral glucose tolerance test (OGTT) was performed. The mice were orally gavaged with d-glucose (2 g/kg body weight), and then blood glucose levels were measured at 15, 30, 60, 90, and 120 min after administration. OGTT result showed that the area under the curve was highest in the HFD group compared to other groups (Fig. 4b, c). Compared to the HFD group, the reduction degree was highest in the CON group with a degree of 50% (*p* = 0.0002), followed by the FH group with a degree of 46% (*p* = 0.0056), the POC group with a degree of 28% (*p* = 0.0448), and FL group with a degree of 20% (*p* = 0.2395). These results demonstrated that *F. venenatum* improved glucose tolerance in obese mice.

Serum samples were collected at week 12 to evaluate lipid profiles, liver toxicity biomarkers, and inflammatory cytokines. The HFD group showed a significantly higher TC level than the CON group at approximately 48% (p < 0.0001). The FL, FH, and POC groups showed significantly lower levels than the HFD group at approximately 22, 29, and 20%, respectively (Fig. 4d) (*p* = 0.0010, *p* < 0.0001, and *p* = 0.0018, respectively). There were no significant differences between the FL, FH, and POC groups. A similar result was observed in the TG (Fig. 4e) (CON, 56%, *p* < 0.0001; FL, 31%, *p* = 0.0011; FH, 67%, *p* < 0.0001; POC, 27%, *p* = 0.0053 compared with the HFD group). There were no significant differences between the CON and FH groups (*p* > 0.9999). At the LDL level, the HFD group was significantly higher compared to the CON group by approximately 67% (*p* < 0.0001). FL, FH, and POC groups were significantly lower than those in the HFD group by approximately 41, 55, and 44%, respectively (Fig. 4f) (*p* = 0.0007, *p* < 0.0001, and *p* = 0.0003, respectively). There were no significant differences between the FL, FH, and POC groups. No significant differences were observed between the CON and FH groups (*p* = 0.7029). HDL levels were significantly lower in the CON group than in the HFD group (Fig. 4g) (*p* = 0.0079). There were no significant differences between the HFD, FL, FH, and POC groups. In ALT and AST levels, the HFD group was significantly higher than the CON group by approximately 57% and 46%, respectively (Fig. 4h, i) (*p* < 0.0001 for ALT and *p* < 0.0001 for AST). The HFD group showed the highest levels of ALT and AST compared to the other groups.

IL-1β level was significantly increased in the HFD group compared to the CON, FL, FH, and POC groups at approximately 45, 53, 43, and 47%, respectively (Fig. 4j) (*p* = 0.0001, *p* < 0.001, *p* = 0.0003, and *p* < 0.0001, respectively). There were no significant differences between the CON, FL, FH, and POC groups. However, IL-10 levels in the HFD group were the lowest among the groups (Fig. 4k). The CON and FH groups were significantly higher than those in the HFD group at approximately 89% and 152%, respectively (*p* = 0.0008 for CON and *p* < 0.0001 for FH). The results revealed that *F. venenatum* improved lipid profile, liver toxicity biomarkers, and inflammatory cytokines.

### White fat mass of obese-induced mice fed *F. venenatum*

**Fig. 5:**
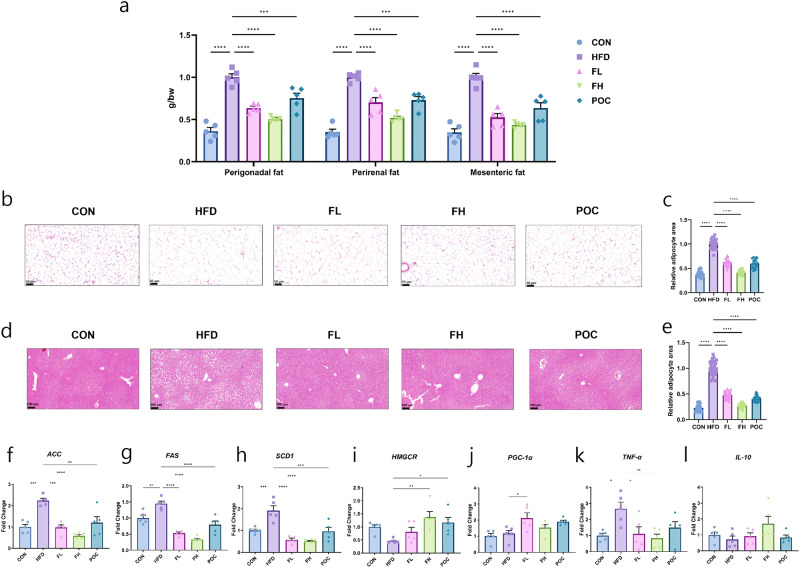
*F. venenatum* reduced fat accumulation in both adipose tissue and liver tissue of high-fat diet-fed mice. **a** Weight of three parts of fat, including perigonadal fat, perirenal fat, and mesenteric fat. **b** Representative images of hematoxylin and eosin-stained adipocytes in mesenteric adipose tissue. Scale bar, 50 µm. **c** Relative adipocyte area in mesenteric adipose tissue. **d** Representative images of hematoxylin and eosin-stained adipocytes in liver tissue. Scale bar, 100 µm. **e** Relative adipocytes area in liver tissue. **f** *ACC*. **g** *FAS*. **h** *SCD1*. **i** *HMGCR*. **j** *PGC-1α*. **k** *TNF-α*. **l** *IL-10*. Data are expressed as means ± SEM. Statistical analysis was performed using One-way ANOVA and differences were considered significant when the *p*-value was below 0.05 (*), 0.01 (**), 0.001 (***), 0.0001 (****). CON group received no oral administration with a normal diet; the HFD group received an oral administration of 200 µL of PBS with a high-fat diet; FL group received an oral administration of 200 mg/kg of *F. venenatum* with a high-fat diet; FH group, received an oral administration of 800 mg/kg of *F. venenatum* with a high-fat diet; POC group, received an oral administration of 40 mg/kg of simvastatin with a high-fat diet.

After 12 weeks, white fat mass was measured in three different parts of the adipose tissue (perigonadal fat, perirenal fat, and mesenteric fat) to estimate fat accumulation (perigonadal fat, perirenal fat, and mesenteric fat) (Fig. 5a). In perigonadal fat, the HFD group showed approximately 64% higher fat mass than the CON group (*p* < 0.0001). Compared to the HFD group, the fat depot was significantly reduced in the FL, FH, and POC groups by approximately 36%, 50%, and 25%, respectively (*p* < 0.0001, *p* < 0.0001, and *p* = 0.0008, respectively). In perirenal fat, the HFD group showed approximately 65% higher fat depots than the CON group (*p* < 0.0001). Compared to the HFD group, fat accumulation was significantly lower in FL, FH, and POC groups by approximately 30%, 48%, and 27%, respectively (*p* < 0.0001, *p* < 0.0001, and *p* = 0.0007, respectively). In mesenteric fat, the HFD group showed approximately 65% higher fat deposition than the CON group (*p* < 0.0001). Compared to the HFD group, fat accumulation was significantly reduced in the FL, FH, and POC groups by approximately 48%, 57%, and 37%, respectively (*p* < 0.0001, *p* < 0.0001, and *p* = 0.0006, respectively). Additionally, histological evaluation in mesenteric adipose tissue indicated that the CON, FL, FH, and POC groups showed significantly reduced relative adipocyte area than the HFD group at approximately 61, 37, 59, and 40%, respectively (Fig. 5b, c) (*p* < 0.0001, *p* < 0.0001, *p* < 0.0001, and *p* < 0.0001, respectively). There were no significant differences between the CON and FH groups (*p* = 0.8384). These results indicate that *F. venenatum* alleviates fat accumulation in three different parts of adipose tissue.

### Hepatic lipid metabolism of obese-induced mice fed *F. venenatum*

Histological evaluation of fat accumulation was also performed in the liver. The CON, FL, FH, and POC groups showed significantly reduced relative adipocyte area compared to the HFD group at approximately 77, 52, 73, and 59%, respectively (Fig. 5d, e) (*p* < 0.0001, *p* < 0.0001, *p* < 0.0001, and *p* < 0.0001, respectively). There was no significant difference between the CON and FH groups (*p* = 0.4395).

RT-qPCR was conducted to evaluate the altered gene expression related to fat metabolism in the liver. In the liver, the genes related to fatty acid synthesis (*ACC*, *FAS*, and *SCD-1*) were significantly increased in the HFD diet group compared to the other treatments. The FL, FH, and POC groups showed a significantly lower *ACC* level compared to the HFD group at approximately 56, 74, and 46%, respectively (Fig. 5f) (*p* = 0.0001, *p* < 0.0001, and *p* = 0.0013, respectively). Similarly, the level of gene expression of *FAS* in the FL, FH, and POC groups was significantly lower than that in the HFD group by approximately 64, 77, and 45%, respectively (Fig. 5g) (*p* < 0.0001, *p* < 0.0001, and *p* < 0.0001, respectively). The gene expression levels of *SCD1* in the FL, FH, and POC groups were significantly reduced compared with those in the HFD group by approximately 70, 73, and 50%, respectively (Fig. 5h) (*p* < 0.0001, *p* < 0.0001, and *p* = 0.0006, respectively). Gene expression levels of *HMGCR* were significantly higher in the FH and POC groups than in the HFD group at approximately 200% and 155% (*p* = 0.0046 and *p* = 0.0348, respectively), and the FL group showed increased gene expression by approximately 76% compared to the HFD group (Fig. 5i). Dietary supplementation of *F. venenatum* could increase the gene expression level of *PGC-1α* in the FL and FH groups compared to that of HFD group by approximately 82% and 31%, respectively (Fig. 5j). The expression of *TNF- α* was the highest in the HFD group and the level decreased by feeding *F. venenatum* in a dose-dependent manner (Fig. 5k) and the gene expression of *IL-10* showed positively increased tendency in FL and FH groups compared with the HFD group (Fig. 5l). These results indicated that *F. venenatum* can reduce fat accumulation in the liver by decreasing gene expression related to hepatic adipogenesis. In addition, it can decrease gene expression related to pro-inflammatory cytokines while increasing gene expression related to anti-inflammatory cytokines.

### Multi-omics analysis on the gut environment of obese-induced mice fed *F. venenatum*

**Fig. 6:**
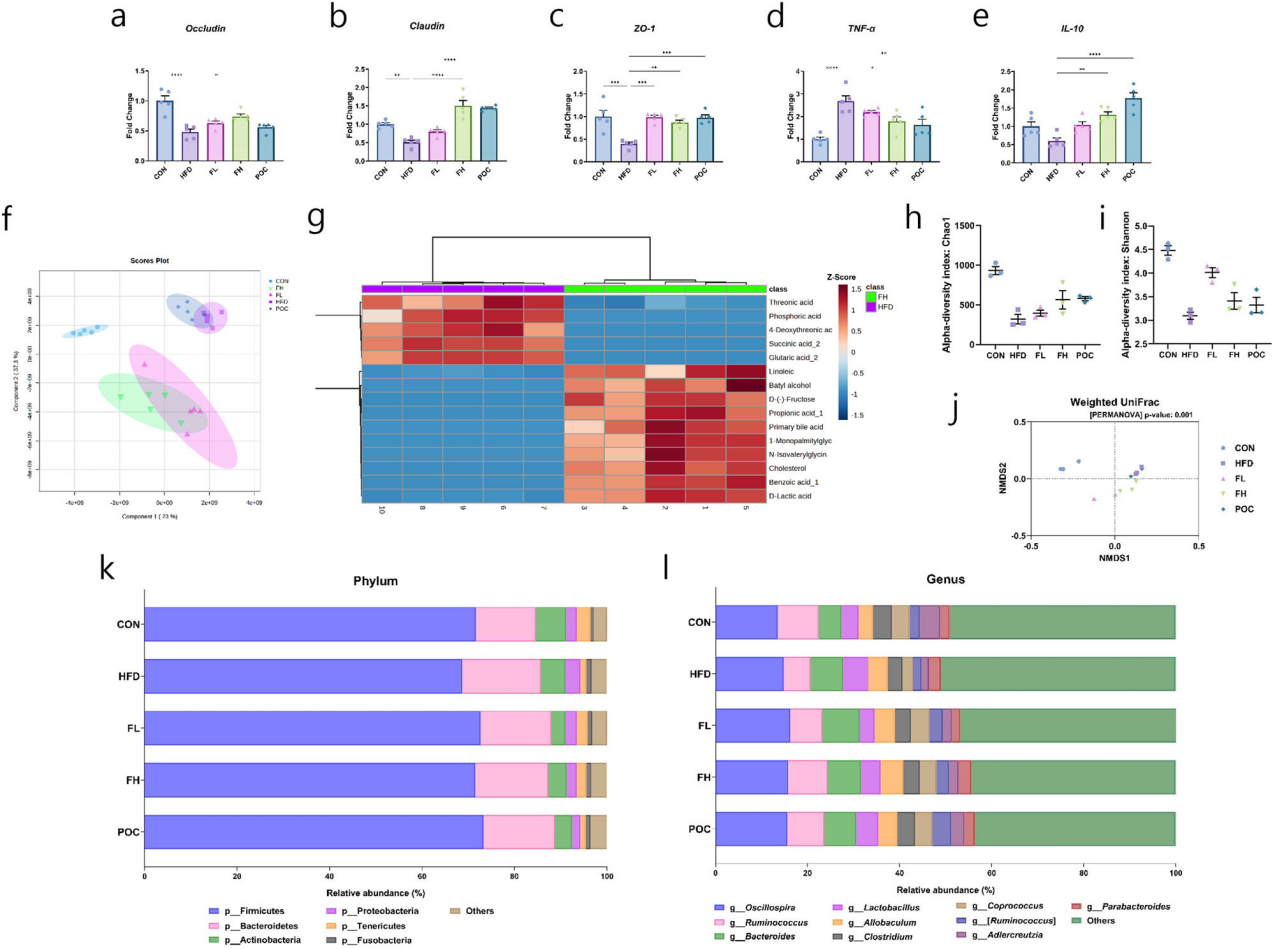
*F. venenatum* entrapped bile acid and cholesterol and then excreted them through feces. **a** *Occludin*. **b** *Claudin*. **c** *ZO-1*. **d** *TNF-α*. **e** *IL-10*. **f** PLS-DA analysis with fecal metabolites of all groups. **g** Heatmap analysis with the top 15 changed fecal metabolites between HFD and FH. **h** Alpha diversity (Chao1 index values) comparison of fecal microbiota among the groups. **i** Alpha diversity (Shannon index values) comparison of fecal microbiota among the groups. **j** PCoA plots based on weighted UniFrac distances of fecal microbiota. Each plot represents each sample, and the axes represent the two dimensions that account for the highest amount of variance in the communities. **k** Microbial composition at the Phylum level. **l** Microbial composition at the genus level. Data are expressed as means ± SEM. Statistical analysis was performed using One-way ANOVA, and differences were considered significant when the *p*-value was below 0.05 (*), 0.01 (**), 0.001 (***), 0.0001 (****). CON group received no oral administration with a normal diet; HFD group received an oral administration of 200 µL of PBS with a high-fat diet; the FL group received an oral administration of 200 mg/kg of *F. venenatum* with a high-fat diet; FH group received an oral administration of 800 mg/kg of *F. venenatum* with a high-fat diet; POC group received an oral administration of 40 mg/kg of simvastatin with a high-fat diet.

In the gut, the HFD group showed a decreased expression level of tight junction-related genes (*Occludin*, *Claudin*, and *ZO-1*) compared with other groups. The level of gene expression of *Occludin* and *Claudin* increased in the groups FL and FH compared with the HFD group by approximately 31 and 54% in *Occludin* and 56 and 190% in *Claudin,* respectively (Fig. 6a, b). Especially in the FH group, the expression level was significantly higher compared with the HFD group (*p* = 0.0138 in *Occludin* and *p* < 0.0001 in *Claudin*). Moreover, the expression level of *ZO-1* was significantly increased in the group FL and FH groups compared with the HFD group by approximately 149% and 121% (*p* = 0.0001 and *p* = 0.0016, respectively) (Fig. 6c). The expression of *TNF- α* was the highest in the HFD group and the level was reduced in groups fed with *F. venenatum* in a dose-dependent manner (Fig. 6d). The gene expression of *IL-10* showed a higher level in FL and FH groups compared with the HFD group as the dose of *F. venenatum* increases (Fig. 6e). These results indicated that *F. venenatum* can reduce gene expression related to pro-inflammatory cytokines while enhancing gut integrity by increasing gene expression related to intestinal tight junction and anti-inflammatory cytokines.

Metabolomic analysis was performed to observe changes in metabolite composition among the groups. The CON group, fed a normal chow diet, formed a separate cluster, while the HFD and POC groups clustered together, and the FL and FH groups clustered together (Fig. 6f). A heatmap was generated with the top 15 significantly altered metabolites between the HFD and FH groups (Fig. 6g). The results showed that primary bile acid and its precursor cholesterol were detected more frequently in the FH group, indicating that *F. venenatum* entrapped primary bile acid and dietary cholesterol and excrete them through feces.

Metagenomic analysis was performed using week 12 fecal samples to evaluate the differences in microbiota composition among groups. The Chao1 and Shannon indexes were used to measure the alpha diversity of fecal samples. The CON group was the highest in the Chao1 index, while the HFD group was the lowest. (Fig. 6h). The Shannon index showed similar results to the Chao1 index (Fig. 6i). The beta diversity data showed that all samples clustered according to their respective groups (Fig. 6j). At the phylum level, Firmicutes were relatively less abundant in the HFD group than in the other groups, while Bacteroidetes, Proteobacteria, and Fusobacteria were relatively abundant in the HFD group (Fig. 6k). At the genus level, the FL, FH, and POC groups showed a relatively high abundance of *Clostridium* and *Coprococcus* compared to the HFD group (Fig. 6l).

## Discussion

The obese population is increasing worldwide, and excessive meat consumption is considered a cause of obesity. Therefore, finding new alternative protein sources that are sustainably supplied and can help improve obesity has become a leading interest in the future. Owing to its high protein, high fiber, low fat, and low energy content, *F. venenatum* has attracted attention as an adequate meat substitute and anti-obesity food^3^.

In the first experiment, we used *C. elegans* as a surrogate animal model to evaluate *F. venenatum* as an alternative protein source and to examine its effects on lipid metabolism. We first evaluated whether *F. venenatum* and the extracted proteins could prolong the lifespan of *C. elegans*. Feeding *F. venenatum* extended longevity in both the L1 and L4 stages of *C. elegans*. In addition, proteins extracted from *F. venenatum* at concentrations of 0.75 and 1 mg/mL extended the lifespan of *C. elegans*. This indicated that *F. venenatum* and its proteins help prolong the longevity of *C. elegans*. One of the possible reasons for the improved lifespan of *C. elegans* induced by *F. venenatum* is the presence of β-glucans^11^.

*C. elegans* has evolved an innate immune response to protect against infection by pathogenic bacteria. We performed a killing assay to determine whether *F. venenatum* could improve the immune response of *C. elegans* against pathogenic bacteria. Preconditioning *C. elegans* with *F. venenatum* showed protective effects against both gram-negative (*S*. *typhimurium* SL1344 and *E. coli* O157:H7 EDL933) and gram-positive (*L. monocytogenes* EGD-e and *S. aureus* Newman) pathogens. These results indicate *F. venenatum* may induce an immune response of *C. elegans* against food-borne pathogenic bacteria. The cell wall of *F. venenatum* is predominantly composed of glucosamine, chitin, and β-glucans^12^. One possible reason for the improved immune response against pathogenic bacteria in *C. elegans* may be attributed to the presence of β-glucans in the cell wall of *F. venenatum*, a natural compound with immunostimulatory and immunomodulatory properties. Further studies are needed to confirm the relationship between the improved immune response and the β-glucan content of *F. venenatum* in *C. elegans*. Moreover, *Fusarium* sp. produces secondary metabolites like antibiotic Y, beauvericin, enniatins, and fusaric acid that possess antibacterial properties^13^. These results may also indicate that *F. venenatum* itself protects *C. elegans* from pathogenic bacteria in the intestine. Further studies are needed to elucidate whether *F. venenatum* exerts antimicrobial activity against intestinal pathogenic bacteria.

Next, we measured the body size of *C. elegans* to evaluate whether *F. venenatum* altered the phenotype of *C. elegans*. We confirmed a reduction in length and width, which may be reflected by reduced fat accumulation. The β-glucans present in the cell wall of *F. venenatum* may be the reason for the reduction in fat accumulation observed in *C. elegans*, since β-glucan extracts from *Phellinus baumii* and *Grifola frondosa* mushrooms also exhibited anti-obesity and anti-aging activities in *C. elegans*^14,15^. Collectively, these results imply that the composition of the cell wall of *F. venenatum* may help reduce fat accumulation in *C. elegans*.

A previous study based on gene expression during fat storage in *C. elegans* reported that numerous gene expressions can affect phenotypes^9^. Therefore, whole-transcriptomic analysis was performed to identify differentially expressed genes related to fat metabolism in *F. venenatum*. We found that *F. venenatum* significantly downregulated genes related to fat synthesis and upregulated those related to the fat breakdown pathway. The fat synthesis pathway starts with acetyl-CoA, the primary substrate. Next, *POD-2* carboxylates acetyl-CoA to create malonyl-CoA, which is then converted by *FASN-1* into fatty acids of varying lengths, primarily palmitic acid (C16:0). These fatty acids are further modified by desaturase or elongase enzymes and form triglycerides, which are then stored^9^. In this study, *F. venenatum* significantly downregulated *POD-2* and *FASN-1* gene expression which agrees with previous studies showing that downregulated expression of *POD-2* and *FASN-1* led to a reduction in fat accumulation^16^. In the fat breakdown pathway, triglycerides are broken down into glycerol and fatty acids. The short-, medium-, and long-chain fatty acids are broken down in mitochondria, and very-long-chain fatty acids are broken down in peroxisomes^9^. In this study, *F. venenatum* significantly upregulated multiple genes related to fat breakdown pathways in both mitochondria and peroxisomes, which may lead to reduced fat storage in *C. elegans*. This was consistent with previous studies indicating that upregulated expression of genes related to fat breakdown pathways can reduce fat accumulation^17^.

Cytoscape was used to determine the significantly altered metabolic pathways in *F. venenatum*. We used significantly upregulated genes to identify the upregulated pathways. The Foxo signaling pathway was upregulated, which is known to increase lifespan in *C. elegans*^18^. In addition, feeding *F. venenatum* up-regulated tryptophan metabolism which, according to a previous study, can increase longevity in *C. elegans*^19^. Also, we found that glutathione metabolism was upregulated, which can prolong lifespan by neutralizing reactive oxygen species as a cellular antioxidant^20^. Significantly downregulated genes were used to confirm the downregulation of the metabolic pathway. We confirmed that the fatty acid biosynthesis pathway was downregulated by feeding *F. venenatum*. Additionally, we observed the downregulation of cysteine and methionine metabolisms. Upregulated cysteine metabolism in mammals has been linked to increased body fat and the risk of obesity in numerous epidemiologic studies^21^. A previous study found that the antidiabetic drug metformin increased lifespan in *C. elegans* by disrupting methionine metabolism^22^. Collectively, the altered gene expression of *F. venenatum* increased longevity and reduced fat accumulation.

GC-MS was performed to determine the changes in metabolites induced by *F. venenatum* and the relationship between altered metabolites and fat metabolism. PLS-DA analysis revealed altered metabolite profiles after feeding *F. venenatum*. Feeding *C. elegans* with *F. venenatum* upregulated several metabolites, including D-xylose, which can reduce body weight and lipid accumulation in mice^23^, 2-aminoadipic acid that activates β3AR signaling to enhance lipolysis and browning of white adipose tissue in mice^24^, alpha-linolenic acid that increased lifespan in *C. elegans*^25^, and nicotinic acid that can synergistically work with leucine to stimulate AMPK and Sirt1 signaling, resulting in reduced fat accumulation and increased lifespan in *C. elegans*^26^. In addition, *F. venenatum* feeding resulted in the upregulation of BCAAs, including leucine, isoleucine, and valine, which are known to play a crucial role in the insulin/IGF-1 signaling pathway and can extend lifespan in *C. elegans*^27^. In summary, feeding *F. venenatum* to *C. elegans* resulted in changes in the metabolite composition, which were related to improved lifespan and reduced fat accumulation.

In the second experiment, C57BL/6 mice were used as an experimental model to assess the anti-obesity effects of *F. venenatum*. At week 12, we observed a significant reduction in body weight among the groups fed *F. venenatum* compared to the HFD group. We did not observe any significant difference in feed and water intake among groups fed with a high-fat diet. To evaluate the potential of *F. venenatum* in ameliorating hyperglycemia due to long-term intake of a high-fat diet, we examined fasting blood glucose levels and conducted an OGTT. Long-term consumption of excessive calories through a high-fat diet can promote de novo lipogenesis and induce hyperglycemia and insulin resistance. At week 12, the HFD group showed hyperglycemia, while the FL and FH groups fed *F. venenatum* showed reduced fasting glucose levels. The HFD group also showed increased glucose tolerance in the OGTT, while glucose tolerance was dose-dependently reduced by feeding *F. venenatum*.

At week 12, serum samples were collected to evaluate the effects of *F. venenatum* on inflammatory cytokines, liver toxicity biomarkers, and lipid profiles. Obesity is known to be characterized by chronic low-grade inflammation. The results revealed that mice fed *F. venenatum* had reduced pro-inflammatory cytokines and increased anti-inflammatory cytokines compared to the HFD group. Additionally, ALT and AST, biomarkers for liver toxicity, were lower in the groups fed *F. venenatum*, suggesting that *F. venenatum* has no toxic effects on the liver. Lipid profile analysis showed that TC, TG, and LDL were lower in the groups fed *F. venenatum* compared to the HFD group, which is consistent with a previous in vivo study by Thomas^7^. However, the exact mechanism of this reduction has not been fully elucidated in the previous study. An in vitro study by Colosimo, suggested that *F. venenatum* may reduce hydrolysis and lipolysis by entrapping digestive enzymes such as amylase, lipase, and bile salts^8^. Fecal metabolomic analysis revealed high levels of primary bile acid in the feces of mice fed *F. venenatum*, indicating that *F. venenatum* may entrap primary bile acid. Interestingly, a high level of cholesterol, which is a precursor of primary bile acid, was also detected in the feces of mice fed *F. venenatum*. We speculated that *F. venenatum* contributed to a decrease in serum TC and LDL levels by excreting primary bile acid and dietary cholesterol in feces, which may have induced the use of endogenous cholesterol to synthesize primary bile acid to maintain a stable level of the bile acid pool. Furthermore, the entrapment of digestive enzymes including primary bile acid, by *F. venenatum* may have reduced lipid digestion, leading to lower TG levels and inhibiting fat accumulation. Further studies are needed to investigate the potential of *F. venenatum* to entrap other digestive enzymes, such as amylase and lipase, in vivo. In addition, the fecal metabolomic analysis showed that mice fed with *F. venenatum* had higher levels of propionic acid and butyric acid in their feces. A previous study has confirmed that the fiber in *F. venenatum* has prebiotic properties that promote the production of propionic acid and butyric acid at the expense of acetic acid^28^. Therefore, we hypothesize that increased levels of these SCFAs may have beneficial effects in increasing intestinal integrity in mice fed with *F.* *venenatum*.

It is well known that the long-term consumption of HFD can lead to fat accumulation in the liver and adipocytes. In this study, the mice groups fed with *F. venenatum* showed reduced weight of white fat adipose tissue and fat depots in the liver and adipose tissue compared to the HFD group despite no significant difference in feed intake. RT-PCR analysis of the liver showed that the expression of lipogenesis-related genes, such as *ACC*, *FAS*, and *SCD1*, decreased in a dose-dependent manner in mice fed *F. venenatum*. In a previous study using mice, the downregulated transcription of lipogenesis-related genes led to reduced fat accumulation^29^. *HMGCR* is a gene that encodes an enzyme called HMG-CoA reductase, which is a key enzyme in the process of making cholesterol in the body. Simvastatin, which is known as HMG-CoA reductase inhibitor, is a drug that inhibits cholesterol biosynthesis by competitively binding to the catalytic domain of HMG-CoA reductase. When there is not enough cholesterol being produced due to simvastatin, *HMGCR* gene expression increases to compensate^30^. In this study, we found that the mRNA abundance of *HMGCR* was increased in the POC group compared to the HFD group due to the cholesterol-lowering effect of simvastatin. Interestingly, we observed that the groups fed *F. venenatum* also showed enhanced mRNA abundance of *HMGCR* compared to the HFD group in a dose-dependent manner. We hypothesize that the increased expression of *HMGCR* may be a compensatory mechanism to maintain normal cholesterol levels due to the large amount of cholesterol excreted in the feces by *F. venenatum*. Furthermore, we found that feeding *F. venenatum* also increased the expression of tight junction-related genes, including *Occludin*, *Claudin*, and *ZO-1* in the gut. This suggests that *F. venenatum* may improve gut integrity, possibly due to the increased production of SCFAs from its fiber content.

At the phylum level, we found that the HFD group had a lower relative abundance of Firmicutes and a higher relative abundance of Bacteroidetes compared to the other groups. This was consistent with a previous study that induced obesity in genetically similar mice by feeding them a high-fat diet for three months, which also showed a lower relative abundance of Firmicutes and a higher relative abundance of Bacteroidetes in the gut microbiota of obese mice^31^. Additionally, previous research has shown that the Western lifestyle, a low-fiber diet, is associated with obesity, reduced bacterial diversity, and an increased relative abundance of Bacteroidetes. In contrast, a non-Western lifestyle a high-fiber diet, are associated with an increased relative abundance of Firmicutes^32^. Therefore, these results suggest that *F. venenatum*, which is a high-fiber diet, may have increased the relative abundance of Firmicutes and decreased the relative abundance of Bacteroidetes.

The relative abundance of Proteobacteria was reduced in the CON, FL, FH, and POC groups compared to that in the HFD group. Previous studies have shown that an increase in the relative abundance of Proteobacteria is a signature of dysbiosis, which has been linked to obesity status of obesity^33^. Collectively, we speculate that *F. venenatum* may have reduced the relative abundance of Proteobacteria, a marker of dysbiosis and obesity status.

At the genus level, the relative abundance of *Clostridium* and *Coprococcus* was relatively abundant in the FL, FH, and POC groups compared to the HFD group. A study using fecal microbiota transplantation showed that the mice transplanted with wild douc microbiota, consisting of bacteria such as *Clostridium* and *Coprococcus*, effectively prevented weight gain on both low- and high-fiber diets^32^. In line with this, our study found that *Clostridium* and *Coprococcus* were relatively abundant in the groups fed *F. venenatum*, which is high in fiber, compared to the HFD group. This suggests that the high fiber content of *F. venenatum* may promote the growth of these genera, which in turn can help prevent weight gain. We also confirmed the increased gastrocnemius muscle in mice fed *F. venenatum* which suggested that *F. venenatum* may also be related to muscle mass development (Suppl Fig. 1). Further studies are needed to elucidate the effect of *F. venenatum* on muscle development and its potential as a treatment for sarcopenic obesity.

In conclusion, the present study using *C. elegans* suggested that *F. venenatum* can prolong the lifespan, enhance immune response, and reduce fat accumulation. In addition, *F. venenatum* downregulated the expression of genes related to fat synthesis. The results of high-fat diet-induced obese mice showed that *F. venenatum* reduced fat accumulation in the liver and adipose tissue and decreased the expression of genes involved in fat synthesis in the liver. Moreover, *F. venenatum* entrapped digestive enzymes such as primary bile acid and cholesterol, leading to their excretion in feces, which resulted in decreased lipid digestion and cholesterol levels. Collectively, these results indicated that *F. venenatum*-based microbial protein is a potential alternative protein source and functional food to treat obesity. To our knowledge, this report is the first to describe specific mechanisms for the anti-obesity activity of *Fusarium venenatum*-based microbial protein in diet-induced obesity animal models.

## Methods

### Microbial protein and mycotoxin quantification assay

*F. venenatum*-based microbial protein used in this study was produced with *F. venenatum* KACC No.49797 (strain A3/5), which is the same strain that is used for producing Quorn^TM^ products. Fermentation followed the Quorn^TM^ production methods described in previous studies by Finnigan^12^ and Wiebe^34^. The malt extract broth medium (BD Biosciences, Sparks, MD, USA) was used, and the heat treatment process was included to eliminate any potential risks associated with consuming live fungi. Protein extraction from fungi was followed by the method in Bridge^35^. For the mycotoxin quantification assay, zearalenone, fumonisin B1, and fumonisin B2 were measured using high-performance liquid chromatography-tandem mass spectrometry (HPLC-MS/MS) according to the method of Rauová^36^. In addition, deoxynivalenol was quantified using the HPLC-ultraviolet (UV) method according to Klötzel’s method^37^.

### Bacterial strains and culture conditions

*Salmonella* Typhimurium SL1344 was grown at 37 °C for 24 h in a nutrient broth medium (BD Biosciences, Sparks, MD, USA). *Staphylococcus aureus* Newman and *Listeria monocytogenes *EGD-e were grown at 37 °C for 24 h in brain heart infusion (BHI) broth medium (BD Biosciences, Sparks, MD, USA). *E. coli* O157:H7 EDL933 and *E. coli* OP50 were grown at 37 °C for 24 h in Luria–Bertani broth medium (BD Biosciences, Sparks, MD, USA). Then, these bacteria were plated on nematode growth medium (NGM) plates.

### *C. elegans* culture condition

The *C. elegans* strain *fer-15;ferm-1* mutant was used, and they were routinely maintained on NGM agar plates. Eggs were extracted in sodium hypochlorite–sodium hydroxide solution, and synchronized L1 worms were grown on NGM agar plates seeded with *E. coli* OP50 at 25 °C to obtain L4/young adult worms.

### *C. elegans* lifespan and killing assays

To perform a lifespan assay with *F. venenatum*, the L1 or L4 stage of *C. elegans* strain *fer-15;fem-1* were individually transferred with a platinum wire onto 60-mm-diameter NGM agar plates^38^. The control group, referred to as the OP50 group, was administered *E. coli* OP50 (8.0 × 10^9^ colony-forming units/mL (CFU/mL)), and the experimental group, referred to as F.V group, was administered *E. coli* OP50 + *F. venenatum* (20 mg/mL). For lifespan assay, 300 worms per treatment were assayed on three plates (100 worms per plate) and incubated at 25 °C. Next, a lifespan assay with total protein extracted from *F. venenatum* was performed. The protein extracted from *F. venenatum* was prepared at three concentrations (0.5, 0.75, and 1 mg/mL). Treatment groups consisted as follows: NGM agar with *E. coli* OP50 (8.0 × 10^9^ CFU/mL) or *E. coli* OP50 + extracted proteins (0.5, 0.75, 1 mg/mL). For lifespan assay, 90 worms per treatment were assayed on three plates (30 worms per plate) and incubated at 25 °C. The number of live *C. elegans* was counted daily and transferred to a new plate every 2 d. Live or dead *C. elegans* were determined by gently touching a platinum wire, and the assay was conducted until all *C. elegans* died.

To perform the killing assay, L4 stage *C. elegans* were placed onto 35-mm-diameter NGM agar plates with *E. coli* OP50 (8.0 × 10^9^ CFU/mL) or *E. coli* OP50 + *F. venenatum* (20 mg/mL). After preconditioning for 48 h, transferred to NGM agar plates containing pathogenic bacteria, including *S*. Typhimurium SL1344 (8.0 × 10^9^ CFU/mL), *E. coli* O157:H7 EDL933 (8.0 × 10^9^ CFU/mL)*, L. monocytogenes* EGD-e (8.0 × 10^9^ CFU/mL), and *S. aureus* Newman (8.0 × 10^9^ CFU/mL) at 25 °C. For each killing assay, 90 worms per treatment were conducted on three plates (30 worms per plate) and incubated at 25 °C. Live *C. elegans* were measured daily and transferred to a new plate every 2 d. *C. elegans* were gently touched with a platinum wire to determine whether they were alive or dead. The assay was conducted until all *C. elegans* had died.

### *C. elegans* behavior and body size

Locomotive activity and body size were measured using WormLab software (MBF Bioscience, Vermont, USA), as previously reported^39^. Briefly, after preconditioning for 48 h with *E. coli* OP50 or *E. coli* OP50 + *F. venenatum* (20 mg/mL), *C. elegans* were transferred to fresh low-peptone NGM plates seeded with *E. coli* OP50 and allowed to acclimate for 10 min before filming. Each video was recorded for 1 min for tracking analysis. The width, length, and locomotive activity (peristaltic speed [µm/s]) were measured. Ten worms in each group were measured, and the experiments were repeated in triplicate. The pharyngeal pumping rate was measured to estimate feed intake using a stereomicroscope. The pumping rate was monitored by counting pharyngeal contractions for 30 s. At least ten worms in each group were measured, and the experiments were repeated in triplicate.

### Nile red and Oil Red O staining

Briefly, after *C. elegans* reached the L4 stage, it was transferred to plates with *E. coli* OP50 or *E. coli* OP50 + *F. venenatum* (20 mg/mL). After 48 h, *C. elegans* were collected with PBST (1× phosphate-buffered saline + 0.01% Triton X-100). Then the Nile red and Oil Red O staining was performed according to Eacorcia’s method and Yen’s method, respectively^40,41^. Worms were placed on a microscope slide with 2% agarose gel, and 30 worms in each group were photographed using an Olympus IX53 microscope (Olympus, Tokyo, Japan). Images were analyzed using ImageJ software.

### Animals and diets

All experimental protocols and animals were approved by the Institutional Animal Care and Use Committee of Seoul National University (certificate SNU-220111-4). In the present study, 5-week-old male C57BL/6 mice (*n* = 25) were obtained from SamTako Bio (Korea), and five mice were housed in each cage. A normal chow diet and sterile water were provided to mice *ad libitum* under standard laboratory conditions (steady temperature, 23 ± 1 °C; humidity, 55 ± 5%; and 12 h light/dark cycle). After a week adaptation period, mice were randomly divided into five groups of five animals each, depending on body weight (CON, normal chow diet + no oral administration (OA); HFD, high-fat diet + 200 µL PBS OA; FL, high-fat diet + 200 mg/kg *F. venenatum* OA; FH, high-fat diet + 800 mg/kg *F. venenatum* OA; POC, high-fat diet + 40 mg/kg simvastatin OA). The high-fat diet was a 45 kcal%-fat rodent diet (D12451, Research Diets Inc., USA). Body weight, feed intake, and water intake were measured weekly, and all mice were humanely euthanized after 12 weeks. Following euthanasia, we measured the weights of perigonadal fat, perirenal fat, mesenteric fat, and gastrocnemius muscle. These values were then normalized by individual body weight.

### Fasting blood glucose and oral glucose tolerance test (OGTT)

At week 12, the mice fasted for 16 h, and the OGTT was performed. Briefly, D-glucose (2 g/kg body weight) was administered through gavage and blood samples were taken from the tail vein (0, 15, 30, 60, 90, and 120 min after administration) to measure glucose concentration using an Accu-Chek glucose meter (Roche Diagnostics GmbH, Mannheim, Germany).

### Serum analysis

Serum samples were collected at week 12, and biochemical nutritional markers TC, TG, HDL, alanine transaminase (ALT), aspartate transaminase (AST), and inflammatory cytokines (IL-1β and IL-10) were measured. Biochemical, and nutritional markers were measured using a Fuji DRI-CHEM Clinical Chemistry Analyzer FDC 3500 (Fujifilm, Tokyo, Japan), and LDL level was calculated using the Friedewald formula^42^. Serum inflammatory cytokines were measured by enzyme-linked immunosorbent assay (ELISA) kits (IL-1β, Abcam, ab197742; IL-10, Abcam, ab255729). Experiments were performed in accordance with the manufacturer’s instructions. Optical densities were measured at 450 nm using a spectrophotometer (SpectraMax ABS Plus, San Jose, CA, USA).

### Histological analysis

Mesenteric fat and liver samples from each mouse were washed with sterile PBS. Samples were fixed in 10% v/v formalin and embedded in paraffin for hematoxylin and eosin (H&E) staining. The stained samples were observed with a KFBIO digital slide scanner (Konfoong Bioinformation Technology Co., Ltd.). SABIA software (EBIOGEN, Seoul, Korea) was used for the quantification assay.

### Reverse transcription-polymerase chain reaction (RT-qPCR)

Total RNA was extracted from the liver tissues using an RNeasy kit (Qiagen). Total RNA was reverse transcribed into cDNA using an iScript cDNA Synthesis Kit (Bio-Rad). cDNA was amplified on a CFX96™ real-time PCR system (Bio-Rad) using SsoAdvanced™ Universal SYBR® Green Supermix (Bio-Rad). In liver tissue, acetyl-CoA carboxylase (*ACC*), fatty acid synthase (*FAS*), stearoyl-CoA desaturase-1 (*SCD1*), HMG-CoA reductase (*HMGCR*), peroxisome proliferator-activated receptor-α (*PGC-1α*), tumor necrosis factor-α (*TNF- α*), and interleukin-10 (*IL-10*) were measured. In gut tissue *Occludin*, *Claudin*, Zonula occludens-1 (*ZO-1*), *TNF- α*, and *IL-10* were measured. All genes were normalized to GAPDH. Each of the samples was technically replicated twice. Primers used in this study are listed in Supplementary Table 4.

### Transcriptomic analysis

Transcriptomic analysis was performed according to Ryu’s method^43^. Briefly, after a 48 h exposure period, total RNA from worms was immediately extracted to examine gene expression in the host using TRIzol reagent (Invitrogen, Carlsbad, CA, USA) and purified using an RNeasy Mini Kit (QIAGEN, Valencia, CA, USA) according to the manufacturer’s instructions. For RNA-seq, a TruSeq RNA Sample Prep Kit v2 (Illumina, San Diego, CA, USA) was used based on the manufacturer’s instructions, and the cDNA library was constructed based on the basic protocol provided by Illumina. Libraries were then sequenced on an Illumina NovaSeq 6000 platform using paired-end read sequencing (2 × 250 bp). Using Trimmomatic 0.38, the adapter sequence, base quality less than 3 from the ends of the reads, bases not satisfying the window size of 4, and mean quality of 15 were removed. Trimmed data were then generated, with reads shorter than 36 bp removed, and further analysis was performed based on high-quality reads. The index of the reference genome was generated using the Hisat2 v2.1.0 program (https://daehwankimlab.github.io/hisat2/ accessed on November 4, 2020). Next, uniquely mapped reads were quantified using Subread/featureCounts version v1.5.1 (http://subread.sourceforge.net/ accessed on 4 November 2020), using Ensembl version 82 transcriptome definitions. The R package edgeR was used to analyze different expressions between different types of samples in the generated data. To define genes that were significantly differentially expressed, the threshold value of |log2-fold change > 1| and *p*-value < 0.05 were used. To identify the functions of differentially expressed genes, the DAVID online tool and Cytoscape were used.

### Metabolomic analysis

Metabolomic analysis was performed according to Yoo’s method^44^. The worm samples which exposed to treatment for 48 h, and mice fecal samples were collected and stored at −80 °C until metabolomic analysis. Disrupted worms using a pestle (Kontes Glass, Vineland, NJ, USA) and fecal samples were mixed with ice-cold methanol. Then, it vortexed for 1 min and incubated on ice. After centrifugation of the vortexed sample at 10,000 rpm for 10 min at 4 °C, the upper layer of the supernatant was passed with a 0.2 μm pore size polyvinylidene fluoride syringe filter (Whatman, Maidstone, England). The filtered supernatant was concentrated to dryness in a vacuum concentrator and stored at −80 °C prior to derivatization and analysis using GC–MS. The extract was derivatized with 30 µL of a solution of 20 mg/mL methoxyamine hydrochloride in pyridine (Sigma, St. Louis, MO, USA) at 30 °C for 90 min, and 50 µL of N,O-Bis(trimethylsilyl)trifluoroacetamide (Sigma, St. Louis, MO, USA) was subsequently added at 60 °C for 30 min. Fluoranthene was added to the extract as the internal standard.

GC–MS analysis was performed using a Thermo Trace 1310 GC (Waltham, MA, USA) coupled with a Thermo ISQ LT single quadrupole mass spectrometer (Waltham, MA, USA). A DB-5MS column with 60 m length, 0.2 mm i.d., and 0.25 µm film thickness (Agilent, Santa Clara, CA, USA) was used for separation. For analysis, the sample was injected at 300 °C and a split ratio of 1:60 with 7.5 mL/min helium split flow. The metabolites were separated using 1.5 mL constant flow helium with an oven ramp of 50 °C (2 min hold) to 180 °C (8 min hold) at 5 °C/min, to 210 °C at 2.5 °C/min, and to 325 °C (10 min hold) at 5 °C/min. The mass spectra were acquired in a scan range of 35–650 m/z at an acquisition rate of five spectra/s. The ionization mode was subjected to electron impact, and the temperature of the ion source was set to 270 °C. The spectra were processed by the Thermo Xcalibur software using automated peak detection, and the metabolites were identified by matching the mass spectra and retention indices of the NIST Mass spectral search program (version 2.0, Gaithersburg, MD, USA). Metabolite data were normalized based on the intensity of the fluoranthene internal standard. Further analyses were conducted using MetaboAnalyst 5.0 software.

### Metagenomic analysis

Metagenomic analysis was performed following previous study methods^45^. The fecal samples were collected at week 12 and then aseptically homogenized, and gDNA was extracted using the DNeasy PowerSoil Pro Kit (Qiagen, Hilden, Germany). The V4 region of the 16 S rRNA gene was amplified (V4 amplicon primer sequence: forward, 5’-TCGTCGGCAGCGTCAGATGTGT ATAAGAGACAGGTGCCAGCMGCCGCGGTAA-3. ’ reverse, 5’ -GTCTCGTGGGCTCGGAGAT GTGTATAAGAGACAGGGACTACHVGGGTWTCTAAT-3’), and the amplified DNA was sequenced using Illumina® iSeq 100 (Illumina, Inc. San Diego, CA, USA), according to the manufacturer’s instructions. Further analyses were performed using Mothur software and MicrobiomeAnalyst.

### Statistic and reproducibility

In *C. elegans* experiments, we employed different quantities of worms based on the specific assays. For the lifespan assay at the L1 and L4 stages, 300 worms were utilized per treatment. In the lifespan assay using extracted protein and the killing assay using pathogenic bacteria, 90 worms were employed for each treatment. For body size measurement, Nile red and Oil Red O staining, locomotive activity, and pumping rate assessments, 30 worms were used per treatment. In the transcriptomic analysis, approximately 1000 worms were used, while metabolomic analysis involved around 4000 worms. *C. elegans* lifespan and killing assay data were analyzed using the Kaplan-Meier method and graphed SigmaPlot 12.0 (Systat Software Inc.). Other data were statistically analyzed using Prism 9 (GraphPad Software, USA). A total of 25 mice were used in this study. They were divided into 5 groups of 5 mice each. Statistical analysis was performed using Prism 9 (GraphPad Software, USA). Statistical significance was considered when the *p*-values were below 0.05 (*), 0.01 (**), 0.001 (***), and 0.0001 (****). The data in the result section were expressed as the differences between means in percentage, while data in the graph are shown as mean ± standard error of the mean (SEM).

### Reporting summary

Further information on research design is available in the Nature Portfolio Reporting Summary linked to this article.

### Supplementary information

Supplementary Information

Description of Additional Supplementary Files

Supplementary Data 1

Reporting summary

## Data Availability

All data needed to evaluate the conclusions in the paper are present in the manuscript and deposited in the NCBI SRA database with the Bioproject number PRJNA982430. Additional data are available from the authors upon request. The source data behind the graphs in the paper is available in Supplementary Data 1.
