# Supplementary material for: Molecular characterization of Fusarium venenatum-based microbial protein in animal models of obesity using multi-omics analysis
Source: Commun Biol. 2024 Jan 26;7:133. doi: 10.1038/s42003-024-05791-9 (PMC10817893; doi:10.1038/s42003-024-05791-9)
Supplement: Supplementary file 1 — Supplementary Information [file 42003_2024_5791_MOESM1_ESM.pdf]

Supplementary Table

Molecular characterization of *Fusarium venenatum*-based microbial protein in animal models of obesity using multi-omics analysis

Daniel Junpyo Lee[1], An Na Kang[1], Junbeom Lee[1], Min-Jin Kwak[1], Daye Mun[1], Daseul Lee[2], Sangnam Oh [3,*], and Younghoon Kim[1,*]

[1]Department of Agricultural Biotechnology and Research Institute of Agriculture and Life Science, Seoul National University, Seoul 08826, Korea

[2]Agricultural Microbiology Division, Department of Agricultural Biology, National Institute of Agricultural Sciences, Wanju-gun, 55365, Korea

[3]Department of Functional Food and Biotechnology, Jeonju University, Jeonju 55069, Korea

*To whom correspondence should be addressed: osangnam@jj.ac.kr or ykeys2584@snu.ac.kr

Supplementary Table 1. Nutritional composition of *Fusarium venenatum* KACC 49797 (strain A3/5)

**Items**	**Unit**	***F. venenatum* KACC No.49797**
Moisture	g/100g	0.00
Ash		3.24
Crude Protein		22.26
Crude Fat		20.24
Carbohydrate		54.26
Crude Fiber		45.64

Supplementary Table 2. Mycotoxin content of *Fusarium venenatum* KACC 49797 (strain A3/5)

Mycotoxin	Unit	Method	Limit of quantification	Result
Fumonisins B1	µg/kg	HPLC-MS/MS	4	8.60
Fumonisins B2	µg/kg	HPLC-MS/MS	4	Non-detected
Zearalenone	µg/kg	HPLC-MS/MS	5	Non-detected
Deoxynivalenol	mg/kg	HPLC/UV	0.2	Non-detected

Supplementary Table 3. Survival data table of lifespan and killing assay.

	Mean Lifespan (days)	% Increase in Mean Lifespan	Maximum Lifespan (days)	% Increase in Maximum Lifespan	Censorship
**L1 stage**					
**OP50**	9.6 ± 0.1		14 ± 0		
**F.V**	11.6 ± 0.2	20.5 %	17.3 ± 0.9	23.8 %	
**L4 stage**					
**OP50**	8.9 ± 0.1		13.7 ± 0.5		
**F.V**	10.8 ± 0.1	21.4 %	17.3 ± 0.5	26.8 %	1
**Extract protein 0.5 mg/mL**					
**OP50**	6.9 ± 0.2		11.0 ± 0.8		
**F.V**	7.7 ± 0.1	11.7 %	11.0 ± 1.6	0 %	1
**Extract protein 0.75 mg/mL**					
**OP50**	5.7 ± 0.1		11.3 ± 0.9		
**F.V**	7.6 ± 1.0	33.5 %	15.3 ± 0.9	35.3 %	
**Extract protein 1 mg/mL**					
**OP50**	8.9 ± 0.1		12.3 ± 0.5		
**F.V**	11.3 ± 0.2	26.7 %	15.7 ± 0.5	27.0 %	
***S.* Typhimurium SL1344**					
**OP50**	4.7 ± 0.2		9.3 ± 0.9		
**F.V**	5.6 ± 0.2	18.7 %	12.0 ± 0.8	28.6 %	
***E. coli* O157:H7 EDL933**					
**OP50**	5.1 ± 0.4		9.7 ± 0.5		
**F.V**	5.9 ± 0.6	16.6 %	12.0 ± 0.8	24.1 %	
***L. monocytogenes* EGD-e**					
**OP50**	6.3 ± 0.3		9.0 ± 0		
**F.V**	8.1 ± 0.9	27.4 %	12.3 ± 0.9	37.0 %	
***S. aureus* Newman**					
**OP50**	5.3 ± 0.7		9.3 ± 0.9		1
**F.V**	6.2 ± 0.4	16.9 %	11.7 ± 0.5	25.0 %	1

Supplementary Table 4. Primers used in this study. F indicates forward primer and R indicates reverse primer

Genes	Sequence
ACC	F: 5'- GAATCTCCTGGTGACAATGCTTATT -3'
	R: 5'- GGTCTTGCTGAGTTGGGTTAGCT -3'
FAS	F: 5'- CTGAGATCCCAGCACTTCTTGA -3'
	R: 5'- GCCTCCGAAGCCAAATGAG -3'
SCD1	F: 5'- TTCTTGCGATACACTCTGGTGC -3'
	R: 5'- CGGGATTGAATGTTCTTGTCGT -3'
HMGCR	F: 5'- AGCTTGCCCGAATTGTATGTG -3'
	R: 5'- TCTGTTGTGAACCATGTGACTTC -3'
*PGC-1*α	F: 5'- TGTTCCCGATCACCATATTCC -3'
	R: 5'- GGTGTCTGTAGTGGCTTGATTC -3'
Occludin	F: 5'- TCACTTTTCCTGCGGTGACT -3'
	R: 5'- GGGAACGTGGCCGATATAATG -3'
Claudin1	F: 5'- CCTTCGGGAGCTCAGGTGCG-3'
	R: 5'- CCGCGTTGGCCATGGCTCTT -3'
ZO-1	F: 5'- GCTGCCTCGAACCTCTACTC -3'
	R: 5'- TTGCTCATAACTTCGCGGGT -3'
TNF-α	F: 5'- AGGGTCTGGGCCATAGAACT -3'
	R: 5'- CCACCACGCTCTTCTGTCTAC -3'
IL-10	F: 5'- AAGTGATGCCCCAGGCA -3'
	R: 5'- TCTCACCCAGGGAATTCAAA -3'
GAPDH	F: 5'- TGAAGCAGGCATCTGAGGG -3'
	R: 5'- CGAAGGTGGAAGAGTGGGAG -3'
