# Supplementary material for: Molecular characterization of Fusarium venenatum-based microbial protein in animal models of obesity using multi-omics analysis
Source: Commun Biol. 2024 Jan 26;7:133. doi: 10.1038/s42003-024-05791-9 (PMC10817893; doi:10.1038/s42003-024-05791-9)
Supplement: Supplementary file 2 — Description of Additional Supplementary Files [file 42003_2024_5791_MOESM2_ESM.pdf]

Description of Additional Supplementary Files

File Name: Supplementary Data 1
Description: The source data behind the graphs in the paper.
