# Supplementary material for: Molecular characterization of Fusarium venenatum-based microbial protein in animal models of obesity using multi-omics analysis
Source: Commun Biol. 2024 Jan 26;7:133. doi: 10.1038/s42003-024-05791-9 (PMC10817893; doi:10.1038/s42003-024-05791-9)
Supplement: Supplementary file 4 — Reporting summary [file 42003_2024_5791_MOESM4_ESM.pdf]

# nature portfolio

Corresponding author(s): Younghoon Kim
Last updated by author(s): Nov 6, 2023

# Reporting Summary

Nature Portfolio wishes to improve the reproducibility of the work that we publish. This form provides structure for consistency and transparency in reporting. For further information on Nature Portfolio policies, see our Editorial Policies and the Editorial Policy Checklist.

## Statistics

For all statistical analyses, confirm that the following items are present in the figure legend, table legend, main text, or Methods section.

| n/a | Confirmed | |
|---|---|---|
| ☐ | ☒ | The exact sample size (*n*) for each experimental group/condition, given as a discrete number and unit of measurement |
| ☐ | ☒ | A statement on whether measurements were taken from distinct samples or whether the same sample was measured repeatedly |
| ☐ | ☒ | The statistical test(s) used AND whether they are one- or two-sided<br>*Only common tests should be described solely by name; describe more complex techniques in the Methods section.* |
| ☒ | ☐ | A description of all covariates tested |
| ☒ | ☐ | A description of any assumptions or corrections, such as tests of normality and adjustment for multiple comparisons |
| ☐ | ☒ | A full description of the statistical parameters including central tendency (e.g. means) or other basic estimates (e.g. regression coefficient) AND variation (e.g. standard deviation) or associated estimates of uncertainty (e.g. confidence intervals) |
| ☐ | ☒ | For null hypothesis testing, the test statistic (e.g. *F*, *t*, *r*) with confidence intervals, effect sizes, degrees of freedom and *P* value noted<br>*Give P values as exact values whenever suitable.* |
| ☒ | ☐ | For Bayesian analysis, information on the choice of priors and Markov chain Monte Carlo settings |
| ☒ | ☐ | For hierarchical and complex designs, identification of the appropriate level for tests and full reporting of outcomes |
| ☒ | ☐ | Estimates of effect sizes (e.g. Cohen's *d*, Pearson's *r*), indicating how they were calculated |

*Our web collection on statistics for biologists contains articles on many of the points above.*

## Software and code

Policy information about availability of computer code

| | |
|---|---|
| Data collection | ImageJ software<br>WormLab software<br>Olympus IX53 microscope<br>Fuji DRI-CHEM Clinical Chemistry Analyzer FDC 3500<br>SpectraMax ABS Plus<br>KFBIO digital slide scanner<br>SABIA software<br>CFX96 real-time PCR system (Bio-rad)<br>Mothur software<br>MetaboAnalyst 5.0 software<br>MicrobiomeAnalyst 2.0 software |
| Data analysis | SigmaPlot 12.0<br>GraphPad Prism version 9 |

For manuscripts utilizing custom algorithms or software that are central to the research but not yet described in published literature, software must be made available to editors and reviewers. We strongly encourage code deposition in a community repository (e.g. GitHub). See the Nature Portfolio guidelines for submitting code & software for further information.

# Data

Policy information about availability of data

All manuscripts must include a data availability statement. This statement should provide the following information, where applicable:

- Accession codes, unique identifiers, or web links for publicly available datasets
- A description of any restrictions on data availability
- For clinical datasets or third party data, please ensure that the statement adheres to our policy

The metagenomics data generated in this study can be accessed on the NCBI-SRA archive under accession number BioProject PRJNA982430.

# Research involving human participants, their data, or biological material

Policy information about studies with human participants or human data. See also policy information about sex, gender (identity/presentation), and sexual orientation and race, ethnicity and racism.

| | |
|---|---|
| Reporting on sex and gender | n/a |
| Reporting on race, ethnicity, or other socially relevant groupings | n/a |
| Population characteristics | n/a |
| Recruitment | n/a |
| Ethics oversight | n/a |

Note that full information on the approval of the study protocol must also be provided in the manuscript.

# Field-specific reporting

Please select the one below that is the best fit for your research. If you are not sure, read the appropriate sections before making your selection.

☒ Life sciences ☐ Behavioural & social sciences ☐ Ecological, evolutionary & environmental sciences

For a reference copy of the document with all sections, see nature.com/documents/nr-reporting-summary-flat.pdf

# Life sciences study design

All studies must disclose on these points even when the disclosure is negative.

| | |
|---|---|
| Sample size | No statistical methods were conducted to pre-determine the sample size. Sample sizes were chosen based on the previous studies which had shown robust statistical power. |
| Data exclusions | No data were excluded. |
| Replication | A minimum of three biological replicates were used for all assays. The exact number of replicates in independent experiments is mentioned in the manuscript. |
| Randomization | After a week adaptation period, mice were allocated to the experimental groups based on body weight. |
| Blinding | n/a |

# Reporting for specific materials, systems and methods

We require information from authors about some types of materials, experimental systems and methods used in many studies. Here, indicate whether each material, system or method listed is relevant to your study. If you are not sure if a list item applies to your research, read the appropriate section before selecting a response.

## Materials & experimental systems

| n/a | Involved in the study | |
|---|---|---|
| ☐ | ☒ | Antibodies |
| ☒ | ☐ | Eukaryotic cell lines |
| ☒ | ☐ | Palaeontology and archaeology |
| ☐ | ☒ | Animals and other organisms |
| ☒ | ☐ | Clinical data |
| ☒ | ☐ | Dual use research of concern |
| ☒ | ☐ | Plants |

## Methods

| n/a | Involved in the study | |
|---|---|---|
| ☒ | ☐ | ChIP-seq |
| ☒ | ☐ | Flow cytometry |
| ☒ | ☐ | MRI-based neuroimaging |

# Antibodies

| | |
|---|---|
| Antibodies used | Mouse IL-1β ELISA kit, Abcam, ab197742.<br>Mouse IL-10 ELISA kit, Abcam, ab255729. |
| Validation | All primary antibodies used in this study were validated by the manufacturer. Details of the validation statement can be found on the manufacturer's website. |

# Animals and other research organisms

Policy information about studies involving animals; ARRIVE guidelines recommended for reporting animal research, and Sex and Gender in Research

| | |
|---|---|
| Laboratory animals | 5-week-old male C57BL/6 mice were used in this study. Mice had access to food and water ad libitum under standard laboratory conditions (steady temperature, 23 ± 1 °C, humidity, 55 ± 5%, and 12 h light/dark cycle). |
| Wild animals | No wild animals were used in this study. |
| Reporting on sex | Male mice alone were used in our animal experiments. |
| Field-collected samples | No field samples were collected in this study. |
| Ethics oversight | All animal experiments were approved by the Institutional Animal Care and Use Committee of Seoul National University (certificate SNU-220111-4). |

Note that full information on the approval of the study protocol must also be provided in the manuscript.

# Plants

| | |
|---|---|
| Seed stocks | n/a |
| Novel plant genotypes | n/a |
| Authentication | n/a |
